# On the Macroeconomic and Social Impact of the Coronavirus Pandemic in Latin America and the Developing World

**DOI:** 10.1007/s10272-020-0889-x

**Published:** 2020-06-07

**Authors:** Christian R. Proaño

**Affiliations:** Fakultät für Sozial- und Wirtschaftswissens., Otto-Friedrich-Universität Bamberg, Feldkirchenstr. 21, 96045 Bamberg, Germany

## Abstract

While the COVID-19 pandemic posits a significant challenge to all societies around the world, it also reveals in the most dramatic manner the many abysmal differences between so-called advanced economies and the developing world.

The COVID-19 pandemic will undoubtedly be a defining event in years to come both in economic, social and political terms. While the immediate impact of the coronavirus pandemic and the related economic slump was assessed to be of a similar magnitude to that of the Great Recession of 2007-2008 by mid-March 2020, the most recent figures of key variables such as the initial claims on unemployment insurance in the United States or the foreign new orders in Germany indicate that the current drop in economic activity will be rather comparable to the Great Depression of the 1930s.

The social costs triggered by the pandemic and the required social distancing measures including the enforced lockdowns in many countries will be enormous. A surge in domestic violence since the start of the coronavirus lockdowns

**Christian R. Proaño**, University of Bamberg, Germany.

has already been recorded in many countries (WHO, 2020), and other side effects concerning the mental health of the population including a sharp increase in suicide rates can be expected, as research on past economic recessions indicates (Schwandt and von Wachter, 2020). At the same time, the roll-back of some civil rights under the premise of halting the spread of the virus may lead to increased authoritarianism in countries where democratic institutions are already weak, such as Hungary, the Philippines and Brazil.

While the COVID-19 pandemic posits a significant challenge to all societies around the world, it also reveals in the most dramatic manner the many abysmal differences between so-called advanced economies and the developing world. While most advanced economies have been able to readily approve and implement fiscal stimuli of a significant dimension – whether the German initiatives or the $2 trillion US stimulus package – and in France, Germany, Italy, Japan and the United Kingdom public-sector liquidity support programmes each above 10% of the respective GDPs have been announced (International Monetary Fund, 2020, 2), the fiscal space and the general capability of developing and low-income countries to confront the current coronavirus crisis is far more limited.

Given the extreme pace at which the COVID-19 pandemic has unfolded around the world, the macroeconomic stabilisation programmes readily implemented in the developed world and in particular in the European countries had (rightly) a clear national focus. The medium-term recovery policies must however include an important international dimension due to the fact that the COVID-19 pandemic is a global phenomenon. Given its important role not only in economic terms, but also as a beacon of institutional and political stability in an otherwise much more turbulent world, the European Union should lead the way in ensuring international support for the developing world.

### The situation in Latin America as a predictor for the developing world

Latin America may provide insight into the different challenges faced by developing countries and how the EU could best help those countries. After its announcement in China and the recording of the first COVID-19 cases in Italy in early February, the coronavirus pandemic proceeded to appear in Latin America by the end of February in Brazil and has since spread throughout the region quite rapidly, with Brazil, Ecuador and Mexico having the highest numbers of related deaths to date. An analysis of the current situation in the Latin American region is important not only in its own right, but it may serve as well as a predictor of what is to come in the next few months in other parts of the developing world.

Economic growth in Latin America and the Caribbean (LAC) region has stalled in recent times: the period from 2014 to 2019 saw the lowest levels of growth since the 1950s (OECD, 2020, 3). With this near absence of economic dynamism related to the global economic slowdown, the LAC region's many structural problems such as the high economic and social inequality and the lack of health and social inclusion worsened and became blatantly evident again. These developments were further fueled by the Venezuelan migration and refugee crisis that has led a staggering number of more than four million Venezuelans to flee their country from 2015 until 2018 (Bahar and Douglas, 2018), resettling in neighbouring South American countries and exerting a significant impact on the labour markets and already fragile social safety nets of the destination countries.

Against this background and the current and future challenges posited by the COVID-19 pandemic, the region's current fiscal situation is quite discouraging. While in 2008 LAC countries were in a much better position to deal with the negative macroeconomic impact of the Global Financial Crisis due to their solid fiscal positions and relatively low indebtedness level, the region's current fiscal situation with an average overall fiscal balance of about -3% of GDP and an average public debt of 62% of GDP in 2019 will most likely restrain the region's capability to confront the COVID-19 pandemic. Additionally, the recent significant capital outflows from LAC countries and many other emerging and developing economies will further deteriorate the financing conditions for many of these countries, widening the sovereign and corporate spreads between the advanced and the developing world.

The restricted and in many cases almost non-existent fiscal space is not the only reason why we should expect the developing world to be much harder hit by the coronavirus crisis. There are many structural differences that render containment and mitigation policies adopted in the developed world only partially viable and enforceable in the LAC region and in the developing world in general. First and foremost, the existing intensive care capacities, direly needed for the patients' medical treatment against COVID-19, in developing countries are dramatically behind those of advanced economies. Mexico, for example, has about 25 times less intensive care units per 100,000 inhabitants than Germany (1.2 vs. about 30). Further, the health care systems in the LAC region and the great majority of developing countries cover only a fraction of the population (usually those who are regularly employed). Further, as highlighted by the World Health Organization (WHO), "most of the world's health-care systems continue to rely on the most inequitable method for financing health-care services: out-of-pocket payments …. This deprives many families of needed care because they cannot afford it" (2008, 24). As a result, many potentially infected people may postpone looking for professional health assistance until it may be too late. In the meantime, they may act as disease vectors, undermining the containment policies put in place.

The low health status particularly of the poorest of the population may also act as a catalytic factor in the COVID-19 pandemic, increasing its impact on the already fragile health systems. The apocalyptic scenes that occurred in the Ecuadorian city of Guayaquil in April, where the hospitals and crematoriums collapsed against the surge in deaths, may have been the result of a syndemic, i.e. the aggregation of multiple epidemics at the same time or consecutively on a population (BBC, 2020). It is not unlikely that such a syndemic may occur in other parts of the world, as dengue, yellow and Lassa fevers, among others, have been recorded in various parts of the world since the beginning of 2020 (Centers for Disease Control and Prevention, 2020).

Second, the more widespread urbanisation in developing countries, with megacities of a high population density and poor sanitation and health services available (Henderson, 2002), may also make social distancing measures less effective if not factually futile. The dramatic scenes observed in the Ecuadorian port city of Guayaquil may thus be repeated in megacities like New Delhi or Lagos.

Third, in contrast to advanced economies such as the ones in the euro area or in Scandinavia, where relatively generous unemployment benefits are effective mechanisms of aggregate demand stabilisation, unemployment insurance systems exist only in a few countries in the LAC region. Additionally, a large fraction of the economically active population in LAC countries works in the informal sector and has thus no safety net whatsoever. As many of these people live near or even below the subsistence level, they are likely to circumvent or blatantly ignore the containment policies on a day-to-day basis. As Ricardo Hausmann, Director of Harvard's Growth Lab and former Minister of Finance of Venezuela put it: "If people must choose between a 10% chance of dying if they go to work and assured starvation if they stay at home, they are bound to choose work" (2020). Such a behaviour, though understandable and even rational from an individual economic point of view, will be a great obstacle for the effective containment of the epidemic in many countries.

The medium-term consequences of the containment and lockdown measures are not to be forgotten. The low and quite unevenly distributed internet access in the region may lead to long-lasting and pervasive effects of the school closures and the lockdown measures in general and widen the already dramatic economic and social inequality in the region. Lockdowns have much higher economic and social costs in poorer societies.

## Urging the European Union to ensure international support for the developing world

China – and in particular, Alibaba's Jack Ma – have recently donated millions of masks and testing kits to developing countries. Rather than standing behind these initiatives, the EU should assume a leadership role at various fronts. First and foremost, the intensive care capacities in the developing world must be expanded massively. This means that the EU should not limit the export of vital medical equipment as the European Commission did in March (Commission Implementing Regulation, 2020). Instead, it should promote the expansion of production capacities to cover not only the needs of EU countries, but the needs of the rest of the world as well. The Coronavirus Global Response pledging marathon initiated by the EU and its partners on 4 May to collect funds for the development and deployment of diagnostics, treatments and a vaccine is an important initiative in these efforts.

On the economic side, the short-run stabilisation of aggregate demand is imperative. The World Bank and the International Monetary Fund have recognised the need to expand the fiscal space for developing countries, activating credit lines and asking G20 leaders to allow the poorest countries to suspend all repayments of official bilateral loans due to the COVID-19 crisis. Given the historically poor record of the LAC region with regards to efficient fiscal policy (Espino and González Rozada, 2012; Ardanaz and Izquierdo, 2017), financial support from the EU and multilateral institutions should clearly define the permissible use of those funds and monitor their implementation closely.

Obviously, proven macroeconomic stabilisation measures such as Germany's much acclaimed short-term working benefits (*Kurzarbeit*) are not practicable in most LAC economies and the great majority of developing countries. Alternative schemes of social distancing based on cheap and recurrent massive testing may be a feasible strategy with the support of the EU.

However, the medium-run recovery of the developing world will be mostly shaped by their capability to compete and prevail in the post-COVID-19 world. This will be determined by two main factors: access to new and sustainable technology through long-term foreign direct investment and more favourable trade conditions in international markets.

This is not only in the interest of the developing world; it is also in the interest of Europe, the US and the rest of the advanced economies. Macroeconomic stability is a major determinant of political stability, and increasing economic and social inequality leads to more political polarisation (Proaño et al., 2019). Time is quickly running out, and not only for Latin America.
